# Telemedicine and primary care obesity management in rural areas – innovative approach for older adults?

**DOI:** 10.1186/s12877-016-0396-x

**Published:** 2017-01-05

**Authors:** John A. Batsis, Sarah N. Pletcher, James E. Stahl

**Affiliations:** 1 Section of General Internal Medicine, Dartmouth-Hitchcock Medical Center, 1 Medical Center Drive, Lebanon, NH 03756 USA; 2 Geisel School of Medicine at Dartmouth, Hanover, NH USA; 3 The Dartmouth Institute for Health Policy and Clinical Practice, Lebanon, NH USA; 4 Dartmouth Centers for Health and Aging, Dartmouth College, Hanover, NH USA; 5 Health Promotion Research Center at Dartmouth, Lebanon, NH USA; 6 Dartmouth Weight and Wellness Center, Lebanon, NH USA; 7 Centers for Telehealth, Dartmouth-Hitchcock, Lebanon, NH USA

**Keywords:** Obesity, Weight loss, Medicare, Telemedicine, Primary care

## Abstract

**Background:**

The growing prevalence of obesity is paralleling a rise in the older adult population creating an increased risk of functional impairment, nursing home placement and early mortality. The Centers for Medicare and Medicaid recognized the importance of treating obesity and instituted a benefit in primary care settings to encourage intensive behavioral therapy in beneficiaries by primary care clinicians. This benefit covers frequent, brief, clinic visits designed to address older adult obesity.

**Discussion:**

We describe the challenges in the implementation and delivery into real-world settings. The challenges in rural settings that have the fastest growing elderly population, high obesity rates, but also workforce shortages and lack of specialized services are emphasized. The use of Telemedicine has successfully been implemented in other specialties and could be a useful modality in delivering much needed intensive behavioral therapy, particularly in distant, under-resourced environments. This review outlines some of the challenges with the current benefit and proposed solutions in overcoming rural primary care barriers to implementation, including changes in staffing models.

**Conclusions:**

Recommendations to extend the benefit’s coverage to be more inclusive of non-physician team members is needed but also for improvement in reimbursement for telemedicine services for older adults with obesity.

## Background

The growing epidemic of obesity affects over 35% of adults ≥65 years in the United States [1] and is associated with serious health risks [2], including increased risk of functional impairment [3], nursing home admission [4], health care costs [5], and early mortality [6]. The disparities in obesity prevalence are even more apparent in rural areas [7] where older adults are the fastest growing segment of the population at risk for this disease [8]. The degree of excess adiposity is compounded by the onset of age-related loss of both muscle mass and quality, termed sarcopenia, that synergistically further impairs function [9]. Cost-effective strategies are needed to address this problem. Primary care practitioners are the first line for both primary and secondary obesity prevention efforts but must be equipped with adequate tools, training, and capacity to provide appropriate care [10–14]. Large-scale trials such as the Diabetes Prevention Program [15] and the Look-AHEAD [16] trials have been shown to be effective in achieving sustained weight loss, following a frequent, structured behavioral intervention. However, intensive structured behavioral interventions are often impractical to implement in primary care settings due to workforce shortages and lack of specialized resources, and when feasible, the evidence remains weak [17]. Only 12 randomized controlled trials have delivered intensive face-to-face counseling in a recent systematic review [17], yet, none involved the use of primary care practitioners (PCPs). In older adults, even less quality evidence exists with a recent systematic review finding only 6 randomized controlled trials of weight loss interventions, and similarly, none were conducted in a primary care setting [18]. The purpose of this current manuscript is to describe the impact and implementation challenges of the new Medicare Obesity Benefit (MOB) Regulatory Coverage determination in the United States. To our knowledge, little has been written on how it impacts primary care practices and how novel delivery modalities could be integrated to ensure its optimal effectiveness in delivering high quality obesity care.

## Medicare obesity benefit

To encourage PCPs to deliver high quality obesity care in Medicare beneficiaries, the Centers for Medicare and Medicaid Services (CMS) introduced a regulatory coverage benefit in November 2011 [19]. This benefit was designed to reimburse PCPs for delivering up to 22 visits of intensive behavioral therapy (IBT) for weight loss within a primary care setting over a period of 12 months. Beneficiaries are required to lose 3 kg of weight during the first 6 months to remain eligible. Medicare requires the use and documentation of motivational interviewing using the 5 A’s approach (Assess, Advise, Agree, Assist, Arrange) [20] as well as measurement of height and weight at each visit.

In arriving at these regulations, behavioral trials specifically focusing on older adults and weight-loss induced complications specific to this population such as sarcopenia or bone loss [21] were not addressed. CMS recognized the importance of reimbursement in a primary care setting as imperative to its success. The benefit currently covers physicians, associate providers and clinical nurse specialists but is not extended to others integrally involved in behavior change, for example, exercise physiologists, behavioral psychologists, or dieticians. The very slow uptake in practices implementing the MOB is likely due to practice management issues, insufficient reimbursement, the high frequency of visits, and an infrastructure that cannot support this benefit [22]. Our group identified only 46,821 Medicare beneficiaries (0.17%) who availed themselves of the benefit in 2013, accounting for 101,290 claims (mean claims/beneficiary 2.16) [23].

## Implementation challenges of the medicare obesity benefit in rural areas

Despite the availability and lack of utility of the MOB, we are concerned that its implementation within a rural primary care setting poses even more striking challenges. Below we outline some of the major barriers related to the workforce, transportation, and reimbursement issues.

- Workforce shortages & lack of specialized services

To meet growing demand, the United States will need between 20,400-35,000 additional PCPs by 2020 [24]. Most of this demand is in response to gradual population growth and aging [24]. While PCPs must have broad medical expertise, the frequency of delivering such face-to-face visits into an existing, overburdened practice makes implementation of the MOB very difficult. We believe that trying to implement this service under the current primary care workforce structure could potentially result in a real service gap potentially exacerbating existing provider burnout [25–27]. For instance, a PCP working 40 face-to-face hours per week with a patient panel of 2,000 patients may have 600 patients with obesity (~30% obese). Assuming 30% are Medicare beneficiaries (*n* = 180), if 50% fully utilized the benefit (*n* = 90), 29,700 min (495 h or ~62 working days) per year would be needed to deliver the benefit in full. This parallels the time needed for providing other preventive services [28]. Because time is finite, considerable trade-offs in a day-to-day practice would need to occur. To fulfill one benefit, another may be lost as the current workforce and demands suggest that there is not enough provider capacity to meet the demand in primary care [28, 29]. This is exacerbated in rural areas which also lack specialized services in part due to poor recruitment and retention [14]. Lack of nursing staff, dieticians and exercise therapists, all integral parts of the multidisciplinary team are critically needed to deliver comprehensive care [30].

b. Transportation challenges

Transportation and long-distances are barriers to health care services in rural areas [31–34] which leads to higher direct and indirect costs to both the patient and the system [5]. This can create difficult choices where people are prompted to ask their “willingness-to-pay” for the service in either time or money; whether patients will consider the trade-offs to gain the benefits of this service worth the cost [35]. Efficient, rural patient-centered transportation systems are lacking [36] and can lead to considerable stress for patients, families and caregivers. Distances between a patient’s home and primary care practice may be acceptable for occasional, intermittent visits; however long travel distances for a short 15 min MOB visit may be problematic, a barrier that can be compounded by weather-related concerns. Distance is known to be an important factor in seeking healthcare in rural veterans [37] and other populations [38–42], increasing the risk of not returning for care. Inequalities to access to care have even been observed elsewhere, including Japan [43]. Our review of the literature did not find any studies specifically examining a relationship between distance and weight loss or obesity care.

c. Poor Reimbursement mechanisms

As with other venues, reduced reimbursement rates and rising operating costs have forced an increased number of practices to accept fewer Medicare/Medicaid patients creating challenges in access for these at risk populations [44]. The current reimbursement for the G0477 code (the MOB) is $27 per session. We believe that alternative reimbursement models are needed to address this service gap, especially from Medicare, that places value on preventive care and management of chronic illnesses. Whether the current reimbursement system can sufficiently maintain a financial viable practice remains unclear.

## Telemedicine and obesity health promotion

Telemedicine may be a useful modality to overcome a number of these aforementioned barriers. It has the potential to increase delivery of the MOB to a larger number of rural primary care practices, circumventing geographic challenges and patient mobility impediments in older, obese adults. While Telemedicine can be defined broadly, we define it as a remote, two-way, video-conferencing that substitutes for face-to-face visits. An increased reliance on universal technology availability makes Telemedicine a potentially viable option to accommodate visual, physical, hearing and cognitive impairments even in rural, older adults [20, 21]. A number of successful ambulatory services have provided specialist advice using Telemedicine all of which have shown favorable patient outcomes, satisfaction and costs [45–47]. Specific to older adults, much of ambulatory care that has used telemedicine has serviced neurology or psychiatry consultations [48] and focused on the cognitive aspect of the comprehensive geriatric assessment. Telemedicine clinical trials and its research base using older adults is uncommon [49]. With the limited number of PCPs and geriatricians [50, 51], partnering with other providers for a brief clinical interaction without ongoing management may be more practical option.

Surprisingly, few studies have evaluated the use of Telemedicine in delivering weight loss interventions. One group has evaluated weight management delivered using Telemedicine in older adults in the IDEATel project [52]. Subjects were elderly Medicare beneficiaries in rural upstate New York with diabetes, 65.8% of which had a BMI ≥30 kg/m [2]. Each subjects had a televisit with a dietician or nurse educator every 4–6 weeks. The findings noted a reduction in waist circumference of 1.2 cm and an improvement in their diet and exercise knowledge. The authors noted that telemedicine visits could successfully be used to establish behavior change goals [53]. In separate care delivery models, telemedicine has been successful in rural areas using diabetes nurse educators and specialists working with patients and primary care providers [54, 55], even in elderly Medicare beneficiary patients [56]. We advocate further systematic evaluation in broadening telemedicine-based interventions to rural areas that will ascertain overall effectiveness of the intervention.

The majority of Telemedicine obesity studies, to our knowledge, have focused on pediatric populations. A systematic review of pediatric weight management using Telemedicine identified 4 studies and suggested that Telemedicine may be promising for rural families with limited access to treatments. The results were associated with high patient satisfaction [57–60] but no differences in weight or other outcomes [57, 59, 61]. After program implementation, telemedicine increased the number of persons willing to participate in rural areas [61], potentially leading to a substitution of outreach clinics [62]. A separate residential obesity program in obese younger children noted a reduction in BMI with a positive effect on weight reduction and on long-term stabilization of weight [63].

Among the studies conducted in a general adult obesity population, Shaikh’s group used Telemedicine to deliver obesity consultations to rural adults from a University-based center. Results noted improvements in diet, activity levels, and favorable changes in weight [64]. This same study group identified barriers in rural health clinics for delivering obesity interventions. Their findings suggested a lack of local weight management programs, lack of family involvement yet a distinct interest in providing care using Telehealth methods [65]. An evaluation of a home Telehealth program on weight maintenance after a group-based weight loss program did not demonstrate significant differences in weight (0.6 kg in the intervention vs. 0.0 kg in the control), nor did it demonstrate other secondary outcome differences in diet, physical activity, social support or self-efficacy [66]. The successful Veterans Affairs *MOVE! Weight Management program* encountered difficulties when it was implemented in rural environments [67]. This intervention consisted of 12 weekly classes delivered using videoconferencing compared to a control group showing a mean difference of −5.5 ± 2.7 kg (−8.0,-3.0; *p* < 0.001) of weight loss. In Taiwan, a 12-week program of videoconferencing in subjects aged 18–45 years for weight-loss led to a mean weight reduction of 5.9 kg with improvement in metabolic parameters [68]. Telehealth has also been found to be helpful for long-term weight loss maintenance with no significant changes in weight following the initial weight-loss program [69].

## Using telemedicine within the framework of the medicare obesity benefit in rural areas

While future research is needed in older adults, telemedicine potentially can address gaps in primary care service coverage in rural areas to overcome the hurdles of reduced widespread implementation of the MOB.

- Addressing workforce issues and improving access to services:

The maldistribution of the workforce has accounted for, in part, the geographic variation in health outcomes between rural and urban populations [13, 67]. Established teleradiology and tele-ICU have successfully allowed a rapid diagnosis of conditions that smaller critical access hospitals cannot manage. Tele-stroke models that allow distant provider to provide stroke care under the advice of a stroke team have shown improved cost-effective outcomes [70]. Canada has focused on using Telehealth to improve accessibility of health services in remote areas by providing consultations, case discussions and clinical support all of which may increase a clinician’s ability to care for more individuals [71, 72]. Educating PCPs for specific populations can result in improved adherence to evidence-based guidelines and clinical outcomes [71].

Telemedicine can be adapted for obesity care. Traditional obesity counseling is delivered by office-based physicians, who have considerable gaps of knowledge in obesity therapies and management. Furthermore, PCPs have little time to deliver reasonable behavioral strategies to alter nutritional and lifestyle habits. Blanket statements are often the norm and PCPs perceive that a lack of time, educational materials and knowledge account for poor care [73]. One means of task-shifting within busy primary care environments would be to deliver the MOB using other healthcare personnel, including health coaches, dieticians and peers. Despite the potential for cost-savings, improved patient satisfaction, and reduced opportunity costs, many payers still do not reimburse for telemedicine nor do they reimburse for services rendered by allied health staff. This parallels that observed within the structure of the MOB which only permit incident-to billing encounters for non-provider staff, such as counsellors, nurses, or health coaches.

Peer health coaches can extend the capacity of existing practices in providing additional self-managing support for patients. This model has successfully been used in diabetic populations [74]. The Diabetes Prevention Lifestyle program was delivered by lay health educators and conducted in senior centers. This group had significantly greater percent weight loss (3.8% vs. 0.2%), with >38% losing >5% baseline weight [53]. Recognizing the PCPs ability to effect changes in behavior and weight, the *Think Health! Study*, which was based upon the Diabetes Prevention Program, grouped PCP visits with lifestyle coaches every four months. These authors found that 22.5 vs. 10.2% lost weight more than 5% of their baseline weight in the intervention group, suggesting the importance of a moderate-intensity lifestyle coaching to facilitate weight loss [75]. Peer coaching using 12 group and 12 individual contacts demonstrated a mean loss of 5% weight in 27% of African American participants [76]. A pilot evaluation of older adults completing a 14-week intervention demonstrated high retention and participation rates as well as significant improvements in pre- and post- fitness levels based on a peer-mentoring model [77]. Others have pilot tested peer (individual and group) support and showed considerable potential of health coaches providing ongoing support, accountability, and information to support behavioral change. To date, there remains very little in terms of health coaching in the obesity literature.

Dietician-led telemedicine interventions have also been explored [78]. The dietician can be employed by a center that has the capacity to deliver obesity interventions. This is in contrast to rural centers that face recruitment challenges. Dieticians can not only train professionals locally in remote facilities, but can directly provide education to target populations at that site requiring further intervention. In fact, a nutrition telemedicine intervention provided support to patients on the Marshall Islands by remotely demonstrating acceptance and no lack of reservations by patients or providers [78]. Telemedicine can widen the available pool of available providers, not only across the country but across the world.

b. Overcoming transportation barriers

Delivering the MOB in a home-based setting has considerable potential advantages as it minimizes patient travel time, missed work, costs and risks associated with travel. Patients can use existing home-based technologies such as personal computers or tablets and obtain medical care and counseling without the need to leave their homes, reducing the risk for high risk elderly to fall, particular during the winter [79, 80]. Seniors on fixed incomes participating in such programs may reduce the cost of associated travel and other indirect costs. An often overlooked advantage of home medical care practiced through telemedicine is that it may encourage the importance of self-care behaviors, improved compliance to healthcare regimens, improve satisfaction and increased empowerment to patients suffering from chronic illness and diseases allowing them to take an active role in their own care [81, 82]. Aging in place enhances the likelihood to improve one’s health, quality of life and be engaged in their communities, particularly at a time where one has access to such remote technologies in the comfort of one’s home setting [83]. The delivery of MOB using such novel technologies has considerable potential to improve important quality of life and medical outcomes in an older adult population.

c. Broaden reimbursement mechanisms:

Telemedicine may be a reasonable substitute for face-to-face, hands-on encounters for consultation, office visits, individual psychotherapy and pharmacologic management by providing reimbursement at the same rates using a Telehealth modifier GT-via interactive audio and video telecommunications system). The G0477 face-to-face behavioral counseling code for obesity is a covered telemedicine service within a primary care setting [19]. However, all beneficiaries receiving the service must be: i) physically located at an eligible facility located outside of a MSA; ii) the facility must be within a rural health professional shortage area; iii) the facility must be within a rural census tract; iv) the facility must be participating in a Telemedicine demonstration project; v) residing in one of these areas. There is no limitation on the physical location of the health professional delivering the service, although licensing may be a barrier to care. For Medicaid patients, reimbursement of services must satisfy federal requirements of efficiency economy and quality of care. Each state has the flexibility to cover to the extent of the medical care, including the types of telemedicine, where in the state, how it is provided, the types of practitioners and the fees that they will reimburse. The absence of a consistent, comprehensive policy for reimbursement is a serious obstacle to integration of Telehealth.

Home-based video-conferencing care can be a reasonable, cost-effective alternative that we believe should be reimbursable, particularly for rural, older adults. Medicare does not currently reimburse for remote patient monitoring services or services rendered outside of the geographic constraints listed above. We propose that the encounter should be reimbursed according to usual charges if the patient is physically present in their home setting. This may improve patient satisfaction, compliance and reduce overhead in the clinical infrastructure. Transitioning from fee-for-service to value-based payments (non-fee for service models) are needed that favor technologies to improve quality of patient care and reduced costs.

As with the MOB, there are limitations in the types of health professionals that can claim reimbursements for remote services, including physicians, associate providers or clinical nurse specialists. Section 1895(e) of the Act states that Telehealth services are outside the scope of the Medicare home health benefit and home health PPS, hence not providing coverage or payment for services provided via a telecommunications system. We would strongly urge policy makers to allow allied-health professions bill for telemedicine services under the auspices of the MOB.

d. Telemedicine vs. other potential modalities?

A number of other emerging models have demonstrated potential efficacy in achieving weight loss. Befort focused on rural participants using two distinct interventions: one involving a similar intensive face-to-face behavioral framework but included pre-packaged meals using a counsellor, and a second cluster randomized trial of an RN-health delivered phone counseling intervention, the results which have not been published [84, 85]. Others have evaluated differences in telephone counseling vs. in-person care [86, 87], internet-based applications [88, 89], or through a cooperative extension [90]. While each of these have the potential for weight loss, none have been evaluated within a primary care setting infrastructure, nor have they addressed specific rural barriers to transportation and access to care that can be problematic for older adults. Telephone monitoring may be problematic in a population at risk for hearing impairment [91]; older adults often require visualization to make sense of words [91, 92]. The potential effectiveness of telemedicine is unclear but holds potential to overcome specific rural barriers and sensory challenges specific to older adults. Future research needs to evaluate and compare the differences between telemedicine and face-to-face counseling.

## Challenges in telemedicine implementation

We recognize that there are a number of implementation challenges with a telemedicine-delivered MOB (see Table 1), of which we highlight a number of examples below. These can be categorized from an organization, technological, and geriatric-specific level:

- Organizational

Prior to implementation, payors, clinical staff, and patients should be willing to consider such a system by making it a financially viable model for all. First, organizations must be willing to identify that telehealth is a priority in improving access for their patients. Second, practical challenges including overhead costs of the telemedicine infrastructure, including initial set-up and upgrades, at the delivering and receiving ends need to be considered and integrated into a business plan. Third, the organization needs to ensure appropriate billing and capturing of encounters. Low reimbursement continues to be problematic for long-term sustainability of telemedicine systems within a fee-for-service model. A full economic analysis is needed prior to engaging in such a venture. Fourth, for providers that are not familiar with such an infrastructure, continual reassurance is needed that telemedicine is patient-centered, and is associated with high satisfaction similar to face-to-face endeavors [93, 94]. Lastly, all team members should engage in a quality assurance model that provides immediate feedback that ensures continual feedback [62].

b. Technological

While broadband access within rural areas has increased considerably in the past few years, it still lags its urban counterparts [95]. High-speed cable internet, which has greater bandwidth capabilities and a more reliable signal than satellite, may be available at community-based or critical access hospitals, but may not be available with full reliability in small rural clinics or in the homes of patients. In one study in Australia, 25% of all consultations experienced internet connection dropping and sound issues, concluding the need of a bandwidth of at least 512 kbps and a latency of no more than 300 ms to conduct a satisfactory videoconference [96]. Unexpected technical issues arising with implementation should be expected, hence the importance of strong information technology support with continual protocol updating is essential [60]. We recommend thorough testing of equipment before providing service to minimize any IT glitches ensuring seamless delivery of care.

c. Geriatric-specific

Lack of technological savviness can be problematic for older adults that have not had the experience with modern technological devices. Older adults are the fastest growing population using eHealth devices [95] yet may still prefer face-to-face visits [97] making newer conventional models difficult to adopt in this population. Due to the less-personal feel of the telemedicine approach, there are inherent challenges to building rapport with patients which can be perceived as a major barrier to older adults. Geriatricians often perceive non-verbal cues and emotions, which can be inherently difficult in noticing and assessing patients’ emotional reactions via telemedicine [98]. Lastly, older adults often have considerable dexterity and sensory impairments such as hearing and visual impairment [99]. An inability to manipulate the technology without assistance could be problematic, as well as cognitive impairment issues. This latter group could be considerably challenged particularly using a non face-to-face medium. However, there is insufficient evidence of weight loss in this population suggesting that this cohort of patients should be excluded from this mechanism.

**Table 1:** Practice management challenges & proposed recommendations for coverage for medicare obesity benefit in rural areas

	Current state	Barrier	Recommendation
Personnel	Physician, Associate Provider, Clinical Nurse Specialist	Decreased supply of PCPs creating a gap in service coverage	Allowing other healthcare providers or peer-health coaching to deliver service
		Lack of Specialized Services Available	Permit allied health providers in delivering service from larger, specialized centers
Frequency of Visits	22 visits provided by above personnel	Creation of an access issue in practices already overworked and overwhelmed	Maintain visit numbers
			Delegate visits to allied health providers to off-set visits
Clinical Site	Face-to-face or Telemedicine-based office setting	Transportation issues create a barrier to providing health care services	Advocate for Telemedicine in rural areas
		Individuals must resident and receive care in designated service areas	Eliminate requirement of service area
		Home-based care is not covered	Permit home-based care
Reimbursement	G0477 Code - $27/visit	Reduced reimbursement to providers	Increase reimbursements or transition to value-based care model

*PCP* primary care provider

## Additional Considerations

- Rural health promotion

Rural regions need special adaptations of evidence-based behavioral weight control programs. Training of lay persons as a best-practice strategy may improve health promotion efforts within such high-risk communities [100]. Such communities often have sparse healthcare resources yet close knit communities and these social networks may be helpful in disseminating evidence-based interventions [101]. Reports have indicated that adequate social support may lead to improved functional status, particularly in older adults [102, 103]. Peer networks or health coaching have the potential to improve such, despite the paucity of literature on this topic [104]. Health promotion programs have been shown to be effective in community-based settings among older adults engaging in physical activity [78].

b. Group coaching models

Group coaching models have often been used in corporate worlds [105] but little evidence exists within the medical setting. We believe that these models have considerable potential, including: a) a sense that group members share a common struggle and that they want to help each other (altruism); b) positive changes in some individuals can boost self-efficacy in others [106, 107]; c) group coaching may be more readily available across the population allowing for more sustainability; d) making commitments towards goals in front of others leads to a greater sense of responsibility to follow through; e) patients can use other people’s tactics or attempts and apply them to themselves (“willing to try new things’). Group coaching models can be integrated both in community and healthcare settings and are a subject for future investigation.

## Conclusions

The importance and value of the MOB in a primary care setting is evident yet a number of challenges exist that prevent its integration within routine clinical care. Telemedicine can potentially be incorporated to address rural disparities with the engagement of non-physician staff in delivering this benefit remotely but this would require changes by regulatory authorities. There is a paucity of research in telemedicine-specific geriatrics interventions and a need is required to develop pragmatic interventions needed to address this public health challenge.

## Abbreviations

CMS: Centers for medicare and medicaid
IBT: Intensive behavioral therapy
MOB: Medicare obesity benefit
PCP: Primary care provider
